# Evolutionary changes in growth, regrowth and carbohydrate storage in an invasive plant

**DOI:** 10.1038/s41598-018-33218-z

**Published:** 2018-10-08

**Authors:** Tiantian Lin, Peter G. L. Klinkhamer, Klaas Vrieling

**Affiliations:** 1 College of Forestry, Sichuan Agricultural University, 611130 Chengdu, China; 2 Institute of Biology, Section Plant Ecology and Phytochemistry, Leiden University, PO Box 9505, 2300 RA Leiden, The Netherlands

## Abstract

We hypothesized that due to the absence of specialist herbivores in introduced ranges, invasive plants have evolved decreased allocation to carbohydrate storage for regrowth ability and as a consequence allocate more to growth. In this study, we compared plant growth, carbohydrate storage and regrowth ability of invasive and native *Jacobaea vulgaris* in response to complete shoot defoliation. We used invasive *J*. *vulgaris* genotypes from three geographically and climactically distinct regions and compared these with native genotypes from Europe. We found that invasive genotypes initially grew larger while native genotypes regrew larger after defoliation. Before defoliation, the carbohydrate storage in roots of invasive genotypes was 38% lower than native genotypes. Biomass after regrowth increased with root carbohydrate storage while it decreased with structural root mass, showing that it is crucial to study root storage and structural components separately in order to investigate plant regrowth. All studied traits of invasive populations from the three geographically and climatologically distinct regions changed in the same expected direction suggesting that the shifts in herbivore guild were causal to the observed change in growth and regrowth ability rather than environmental factors.

## Introduction

Plant invasions can be considered as large-scale and long-term experiment where major selective forces have changed, and therefore provide an ideal opportunity for ecologists to study evolutionary changes. Upon introduction, the most striking change in the new range is the guild of herbivores attacking the plant. Though still under herbivore pressure by generalist herbivores of the invasive range and occasional specialist herbivores of congeneric plant species, the invasive plant species are freed from their native specialist herbivores^1,2^. Several studies showed that the amount of damage in the invasive range is smaller than in the native range^3,4^. Such a shift to a herbivore guild dominated by generalist herbivores is expected to exert an altered selection on the plants in the invasive range. Since plant defense can be costly^5^ and many defense traits are genetically controlled^6^, an evolutionary change is expected to occur in the invasive genotypes that leads to a decrease in investment in anti-herbivore strategies used to deal with the “old” specialist herbivores. The savings in anti-herbivore investment strategies can be reallocated to plant growth, thus increasing the competitive ability of invasive species over the local plant species and allowing higher seed reproduction as proposed in the Evolution of Increased Competitive Ability (EICA) and Shifting Defence Hypothesis (SDH)^7,8^.

Among plant strategies developed to cope with herbivore pressure, tolerance is an alternative to herbivore deterrence^9–11^. In contrast to resistance and avoidance, tolerance is the aspect of defense which is most poorly understood in plant-herbivore interactions^12^. Herbivore pressure plays a major role in the selection of plants to evolve tolerance. A variety of plants suffer from high levels of mainly specialist herbivory that results in frequent defoliation during their lifetime^13^. The level of tolerance those plants exhibit may vary among sites, reflecting the history of grazing damage, suggesting a strong selection for tolerance^14^. Therefore, in the native range where specialist herbivores are present, the level of tolerance is expected to be higher than in the invasive area where specialist herbivores are absent.

Plant tolerance is considered to be costly because tissue is lost through herbivory. Furthermore, the reserves allocated to tolerance cannot be used for other functions such as survival, growth, or reproduction^12^. Therefore, plant genotypes that have high levels of tolerance are expected to show poorer growth than genotypes with lower tolerance. Although tolerance plays a significant role in the way plants cope with herbivore attack^10^, its evolutionary consequences have rarely been studied within the framework of the EICA and SDH and evidence collected so far is controversial^15–19^. Invasive species that have been freed from their specialist herbivores for many generations are an ideal system to study the selective pressures on tolerance. In this study, we tested the hypothesis that invasive plants have evolved towards a decreased investment in tolerance and an increased investment in growth.

The most common form of plant tolerance to herbivore damage is compensatory regrowth^14^. Many plant species have storage organs located in a relatively safe place, such as belowground roots. This allows that they can tolerate tissue loss to herbivory through compensatory regrowth using these stored reserves^20^. The root system is responsible for the supply of water and inorganic nutrients and has been found to be strongly associated with plant regrowth ability^9,21^.

To be able to regrow after foliar herbivory, plants need carbohydrates to regenerate new leaf tissue after defoliation. Root carbohydrate storage has been reported to play an important role in plant regrowth after defoliation^22–24^. We therefore hypothesize that increased carbohydrate storage leads to increased regrowth ability. Many species rely on having high root/shoot ratios that are speculated to have high levels of storage for regrowth^22,25^. However, roots have multiple functions: nutrient and water supply for growth, structural maintenance of plants, and the storage of resources. Hence, a large root to shoot ratio may not result from high storage levels only, but could also be a consequence of low nutrient availability^26^. Thus, to understand regrowth ability and the role of root size in regrowth, it is essential to study both root storage and root size.

Upon complete defoliation, the root cannot rely on photosynthates from the defoliated shoot while energy is still required to maintain survival of the root cells and functions of the root (e.g. root metabolism and respiration)^27–29^. In the meantime, leaf regeneration requires energy. If the plant is regarded as a balanced system between the roots acquiring nutrients and the shoot supplying energy^26^, complete defoliation leads to an immediate shortage of energy with a surplus of nutrients. At this point, structural root maintenance is competing with plant regrowth for limited energy, since they all draw from the same resource pool. We therefore hypothesize that maintaining a large structural root system is costly and negatively associated with plant regrowth ability.

Along with the release from specialist herbivores, local abiotic factors could also contribute to the evolutionary changes in allocation patterns of invasive plants^2,30,31^. Local climatic conditions in the invasive range are considered as important factors in the post-invasion evolutionary change^32–34^. Potentially adaptations to climatic conditions may interact with adaptations to changes in herbivory. For example, storage in the roots may be associated with higher winter survival^35^. In order to rule out the effect of climatological changes, we studied a system in which multiple invasive regions that differ in climatological conditions are compared. We expect parallel evolutionary changes in the allocation to growth, carbohydrate storage, and regrowth in each of the different geographical and climatological invasive ranges if the shift in the herbivore guild is the main selective force.

In this study, we used *Jacobaea vulgaris* (Common ragwort) as our study species. It is formerly known as *Senecio jacobaea*, a monocarpic perennial plant of the family *Asteraceae*. It is native to Eurasia and was introduced into parts of Australia^36^, New Zealand^37^, Eastern North America and Western North America approximately 110 to 160 years ago^38^. Populations of *J*. *vulgaris* from Western and Eastern North America are geographically isolated. The level of genetic variation of native *J*. *vulgaris* does not differ from invasive *J*. *vulgaris*, suggesting that introductions from multiple source populations have occurred^39^. Doorduin *et al*. (2010) showed that populations from the western coast of Europe are the most likely source populations.

*Jacobaea* *vulgaris* has received pest status in invasive ranges because it has caused significant livestock losses due to its toxic pyrrolizidine alkaloids^40^. In the UK (native range), even *J*. *vulgaris* is attacked by more than 70 herbivores, the most herbivory it suffers is due to two specialist herbivores: *Tyria jacobaeae* (Cinnabar moth) and *Longitarsus jacobaeae* (a flea beetle)^36,41,42^. In Northwestern Europe, especially in dune populations, *J*. *vulgaris* suffers from complete defoliation every 2 or 3 years in mid-June by *T*. *jacobaeae* larvae^41,43^. This defoliation is followed by *L*. *jacobaeae* herbivory in August that also causes leaf loss^42,43^. Native *J*. *vulgaris* plants show strong regrowth after complete defoliation^9,44^. In the USA, an invasive range, *J*. *vulgaris* has been observed to be fed on by more than 40 species of generalist arthropods, but no specialists^1^.

In the past 50 years, *T*. *jacobaeae* and *L*. *jacobaeae* have been introduced as biological control agents into some regions of the invasive range^44–46^. Rapo, *et al*.^47^ tested if an evolutionary change occurred in the invasive range of *J*. *vulgaris* after the exposure to *L*. *jacobaeae* as a biological control agent. However, no signs of adaptation to this specialist herbivore were detected in the invasive range.

In this study, we hypothesized that invasive *J*. *vulgaris* genotypes have evolved: (1) an increased growth ability, (2) a decreased root carbohydrate storage, and (3) a decreased regrowth ability as a response to a change in the herbivore guild in the introduced range. Furthermore, plant regrowth ability is hypothesized to be positively associated with root carbohydrate storage and negatively associated with structural root mass. We compared growth, regrowth and root carbohydrate storage between native and invasive *J*. *vulgaris* before and after artificial defoliation. To our knowledge, this study is one of the first studies to focus on the comparison of regrowth ability and root carbohydrate storage between the same plant species from invasive and native ranges. Hence, the results contribute to the critical evaluation of the role of carbohydrate storage and regrowth in evolutionary shifts that mediate invasion success in plants.

## Results

### Climatic conditions

**Figure 1:**
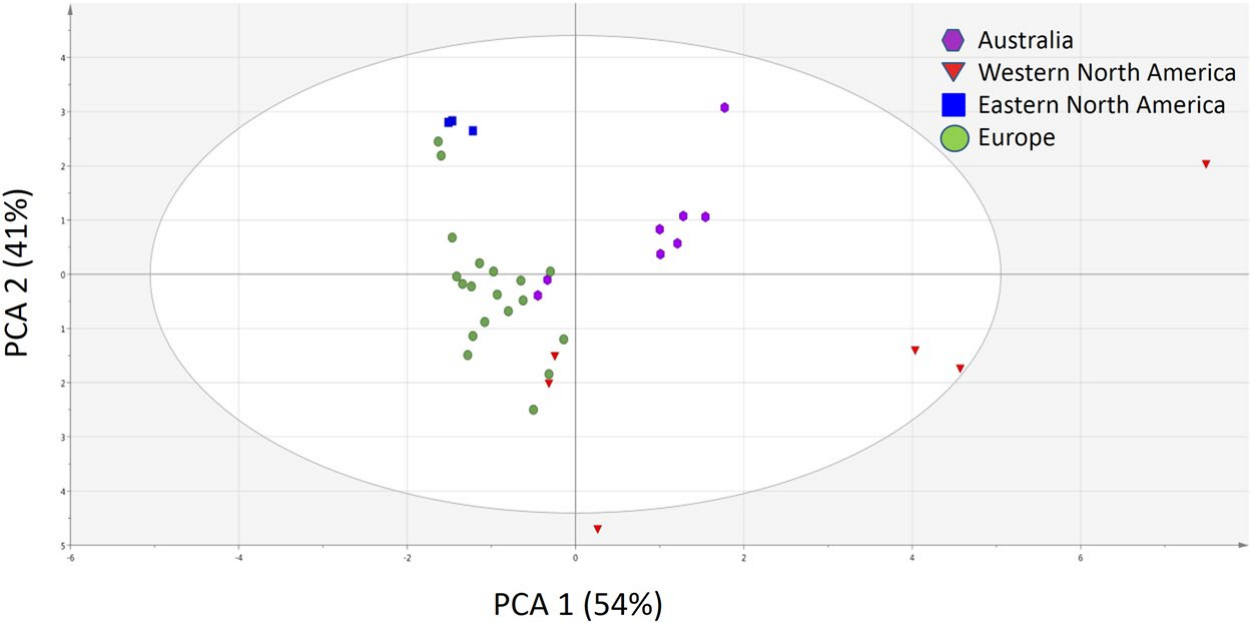
Partial least square-discrimination analysis (PLS-DA) plots for the classification of the four geographic regions based on 19 bioclimatic variables from each of the sampled J. vulgaris populations (N = 18 for Europe, N = 9 for Australia, N = 6 for Western North America and N = 3 for Eastern North America).

Climatic conditions of the sampled invasive *J*. *vulgaris* populations from the three geographically distinct regions (Australia, Eastern North America and Western North America) were separated from each other and the native region except two samples from Australia and two samples from Western North America were mixed with the native region (Fig. 1). We also correlated inulin content with mean temperature of the coldest quarter in the areas of the populations where the seeds were sampled. While native and invasive range did not differ in mean temperature of coldest quarter (ANOVA, F_1,34_ = 0.074, NS), inulin content was not significantly correlated with mean temperature of coldest quarter in the invasive region (r = 0.227, n = 18, NS) and in the native region (r = 0.327, n = 18, NS).

### Initial seedling mass (ControlT0)

**Figure 2:**
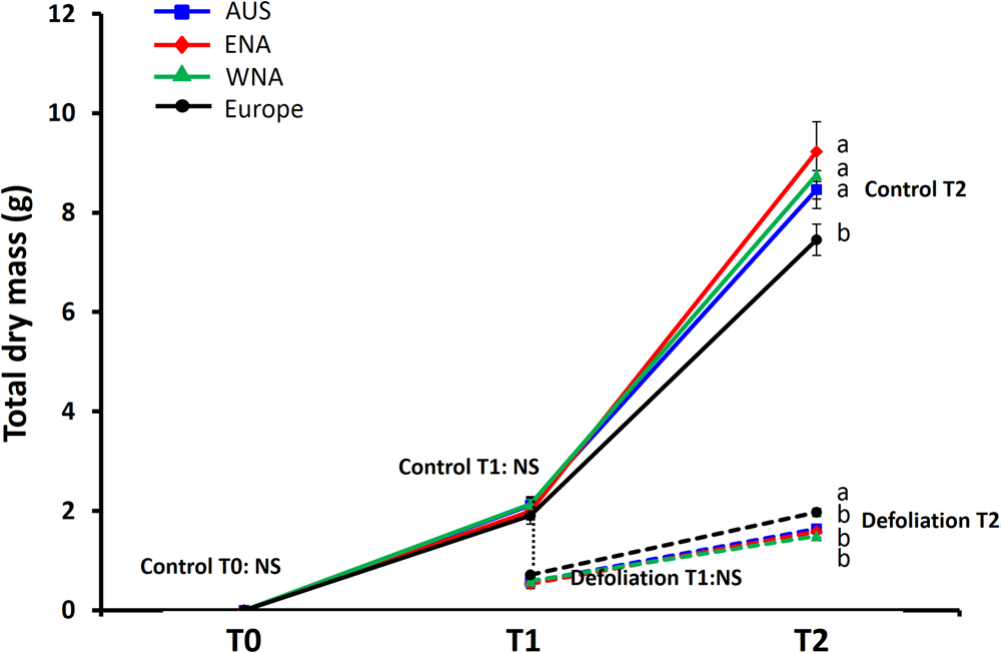
Total dry mass of Jacobaea vulgaris among invasive populations from three geographic regions (Australia, Eastern North America and Western North America) and native populations from Europe at control T0, T1, and T2 and at the defoliation treatment T1 and T2. Solid lines represent the control treatment and dashed lines represent the defoliation treatment. Defoliation T1 is the root dry mass of control T1. Letters indicate significant differences among regions with a Tukey HSD post-hoc test of a nested ANOVA with region as factor and population nested within region, NS = P (region) is not significant.

At the start of the experiment, there was no difference in the dry mass of *J*. *vulgaris* seedlings among the native and three invasive regions (Fig. 2, control T0, nested ANOVA: F_1,3_ = 0.524, p_(Region)_ = 0.669, F_1,32_ = 1.673, p _(Population)_ = 0.037).

### Growth and carbohydrate storage of plants at the first harvest (Control T1)

After 8 weeks of growth, the invasive *J*. *vulgaris* genotypes from the three regions had, on average, a 50% higher total leaf area, a 31% heavier shoot, and a 20% smaller root than their native congeners in the control T1 (Table 1 and Fig. 2). No differences were found in the total dry mass and the structural root dry mass of invasive and native genotypes among the four regions. The invasive genotypes from the three regions had, on average, a 35% higher shoot-structural root ratio than native genotypes. The concentration and content of root inulin of invasive *J*. *vulgaris* genotypes from the three regions were, on average, 25% and 38% lower than native *J*. *vulgaris*, resulting in a root inulin-structural root ratio that was 36% below that of native *J*. *vulgaris* (Table 1). As should be the case, the shoot dry mass removed at defoliation T1 was similar to the shoot dry mass of the harvested plants at control T1 for both native and invasive genotypes (ANOVA, native: F_1,32_ = 0.124, p > 0.05; invasive: F_1,32_ = 0.146, p > 0.05).

**Table 1:** Growth and regrowth traits between invasive and native *Jacobaea vulgaris* populations at control T1, control T2 and defoliation T2.

Variables	Regions	Control T1			Control T2			Defoliation T2		
		Mean ± SE	P (region)	P (population)	Mean ± SE	P (region)	P (population)	Mean ± SE	P (region)	P (population)
Total leaf area (cm^2^)	Australia	489.39 ± 25.59 a	<0.001	0.017	1259.70 ± 55.08 a	<0.001	0.858	154.76 ± 9.12 b	NS	0.014
	ENA	443.58 ± 47.72 a			1365.67 ± 87.88 a			144.85 ± 12.58 b		
	WNA	452.19 ± 32.40 a			1314.42 ± 101.96 a			151.46 ± 13.53 b		
	Europe	312.52 ± 11.78 b			902.73 ± 47.77 b			189.21 ± 9.57 a		
Shoot dry mass (g)	Australia	1.63 ± 0.08 a	0.003	NS	6.96 ± 0.32 a	<0.001	NS	0.99 ± 0.04 b	0.01	NS
	ENA	1.46 ± 0.18 ab			7.60 ± 0.59 a			0.96 ± 0.07 b		
	WNA	1.52 ± 0.11 a			7.24 ± 0.37 a			0.94 ± 0.69 b		
	Europe	1.19 ± 0.05 b			5.35 ± 0.25 b			1.22 ± 0.05 a		
Root dry mass (g)	Australia	0.57 ± 0.04 b	NS (0.055)	NS	1.50 ± 0.09 b	0.005	0.033	0.64 ± 0.05 ab	NS	NS
	ENA	0.53 ± 0.09 b			1.62 ± 0.15 b			0.62 ± 0.05 ab		
	WNA	0.58 ± 0.06 ab			1.52 ± 0.14 b			0.56 ± 0.04 b		
	Europe	0.71 ± 0.04 a			2.10 ± 0.10 a			0.74 ± 0.05 a		
Total dry mass (g)	Australia	2.20 ± 0.11 a	NS	NS	8.46 ± 0.38 a	NS (0.079)	NS	1.64 ± 0.08 b	0.025	NS
	ENA	1.99 ± 0.26 ab			9.23 ± 0.60 a			1.58 ± 0.11 b		
	WNA	2.13 ± 0.15 ab			8.75 ± 0.49 a			1.50 ± 0.10 b		
	Europe	1.90 ± 0.08 b			7.45 ± 0.32 b			1.97 ± 0.09 a		
Structural root (g)	Australia	0.37 ± 0.03 a	NS	NS	0.95 ± 0.06 a	NS	NS	0.45 ± 0.03 ab	NS	NS
	ENA	0.31 ± 0.04 a			0.91 ± 0.06 a			0.43 ± 0.04 ab		
	WNA	0.36 ± 0.03 a			0.89 ± 0.09 a			0.40 ± 0.02 b		
	Europe	0.37 ± 0.02 a			1.02 ± 0.05 a			0.50 ± 0.02 a		
Shoot- structural root ratio (g/g)	Australia	4.61 ± 0.21 a	<0.001	0.039	7.86 ± 0.42 a	<0.001	NS	2.34 ± 0.09 a	NS	NS
	ENA	4.73 ± 0.27 a			8.55 ± 0.69 a			2.30 ± 0.16 a		
	WNA	4.39 ± 0.18 a			9.16 ± 0.86 a			2.32 ± 0.10 a		
	Europe	3.36 ± 0.12 b			5.64 ± 0.32 b			2.66 ± 0.14 a		
Root inulin concentration (mg/g)	Australia	327.73 ± 25.35 b	0.002	NS	350.14 ± 31.03 b	0.006	NS	272.81 ± 22.58 a	NS	NS
	ENA	362.97 ± 48.03 ab			420.75 ± 40.46 ab			292.59 ± 37.65 a		
	WNA	338.48 ± 40.77 b			397.54 ± 42.10 b			266.64 ± 26.56 a		
	Europe	452.29 ± 17.73 a			498.69 ± 18.96 a			297.64 ± 15.35 a		
Root inulin content (mg)	Australia	194.28 ± 21.64 b	0.002	NS	557.12 ± 62.94 b	0.001	NS (0.053)	189.49 ± 22.94 ab	NS	NS
	ENA	221.10 ± 60.33 b			719.69 ± 111.21 b			185.97 ± 29.56 ab		
	WNA	223.41 ± 40.04 b			632.44 ± 100.69 b			159.61 ± 26.12 b		
	Europe	339.00 ± 22.64 a			1078.83 ± 71.57 a			246.11 ± 22.40 a		
Root inulin-structural root ratio (g/g)	Australia	0.54 ± 0.06 b	0.001	NS	0.66 ± 0.10 b	NS	NS	0.41 ± 0.04 a	NS	NS
	ENA	0.64 ± 0.12 b			0.80 ± 0.13 ab			0.45 ± 0.08 a		
	WNA	0.61 ± 0.10 b			0.84 ± 0.15 ab			0.39 ± 0.06 a		
	Europe	0.91 ± 0.05 a			1.20 ± 0.14 a			0.46 ± 0.03 a		

P values are from a nested ANOVA, with region as a fixed factor, and population nested within region as random factors. P (regions): significance level of nested ANOVA among regions. P (populations): significance level of nested ANOVA among populations. Values are means ± SE. Different letters after the values in the Mean ± SE columns indicate significant differences among regions at p < 0.05 according to a Tukey HSD post-hoc test with the same nested ANOVA. ENA represents Eastern North America and WNA represents Western North America.

### Growth and carbohydrate storage of control plants at the second harvest (Control T2)

After 12 weeks of growth without defoliation, invasive *J*. *vulgaris* genotypes from the three invasive regions grew, on average, a 44% larger total leaf area, a 34% larger shoot, a 27% smaller root, and a 17% larger total dry mass than native genotypes at Control T2 (Table 1 and Fig. 2). In addition, invasive genotypes from the three regions did not differ in structural root dry mass from native genotypes but they had, on average, a 49% higher shoot-structural root ratio than native genotypes. Invasive genotypes had, on average, a 26% lower root inulin concentration and 45% lower inulin content that resulted in 28% lower root inulin-structural root ratio than native genotypes (Table 1).

### Growth and carbohydrate storage of defoliated plants (Defoliation T2)

In contrast to at control T2, after four weeks of regrowth following defoliation, invasive *J*. *vulgaris* from the three regions had, on average, 20% smaller shoots and a 20% lower total dry mass than the native *J*. *vulgaris* (Table 1 and Fig. 2). In addition, no clear differences in total leaf area, root dry mass, structural root, shoot-structural root ratio, root inulin concentration, root inulin content and root inulin-structural root ratio were found between defoliated invasive and native genotypes (Table 1).

### Regrowth ability

**Figure 3:**
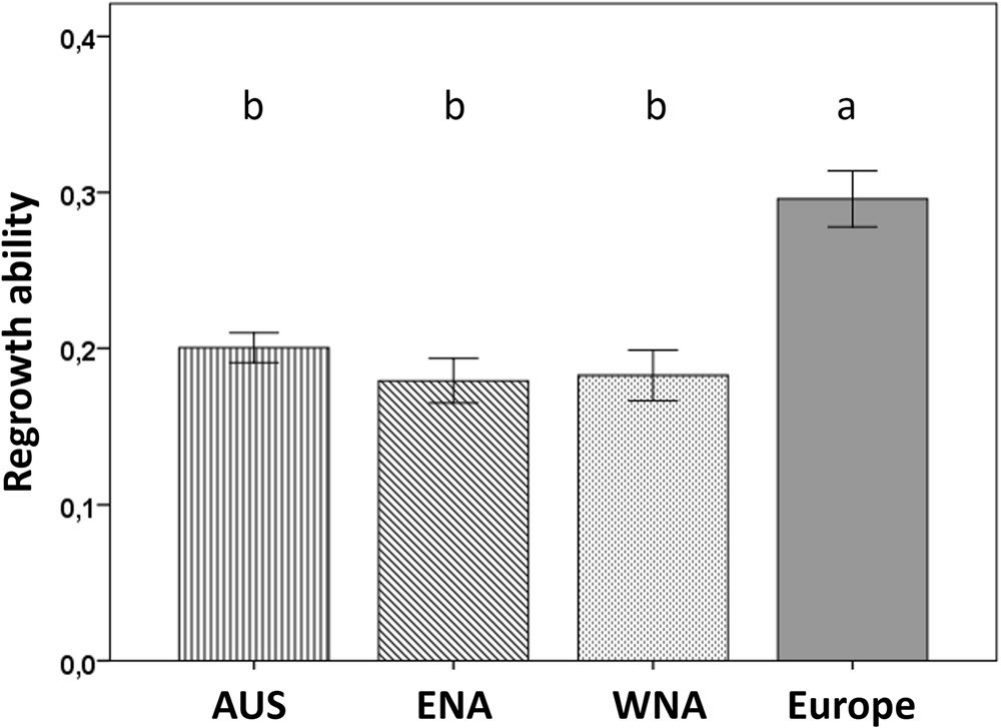
The difference in regrowth ability among invasive populations from three geographic regions (Aus = Australia, ENA = Eastern North America and WNA = Western North America) and native populations from Europe. Values are means ± SE. Letters indicate significant difference s between regions with a Tukey HSD post-hoc test of a nested ANOVA with region as factor and population nested within region (F_1,3_ = 7.198, p (Region) = 0.001, F_1,32_ = 1.247, p (Population) = 0.223).

The regrowth ability was calculated as the ratio between the total dry mass of plants from defoliation T2 and the total dry mass of plants from control T2. The results showed that the invasive genotypes from the three regions had, on average, a 34% lower regrowth ability than native genotypes (Fig. 3).

### Comparison of carbohydrate storage and structural root mass for all treatments

On average, native *J*. *vulgaris* have 62%, 77% and 37% higher root inulin content than invasive genotypes at control T1, control T2 and defoliation T2, respectively (Table 1). Compared to the root inulin content at the moment of defoliation (control T1), the root inulin content of invasive *J*. *vulgaris* genotypes at defoliation T2 was reduced by 14% while in the native genotypes it was reduced by 27%. As for the structural root mass there is no difference between native and invasive genotypes at all the treatments.

**Figure 4:**
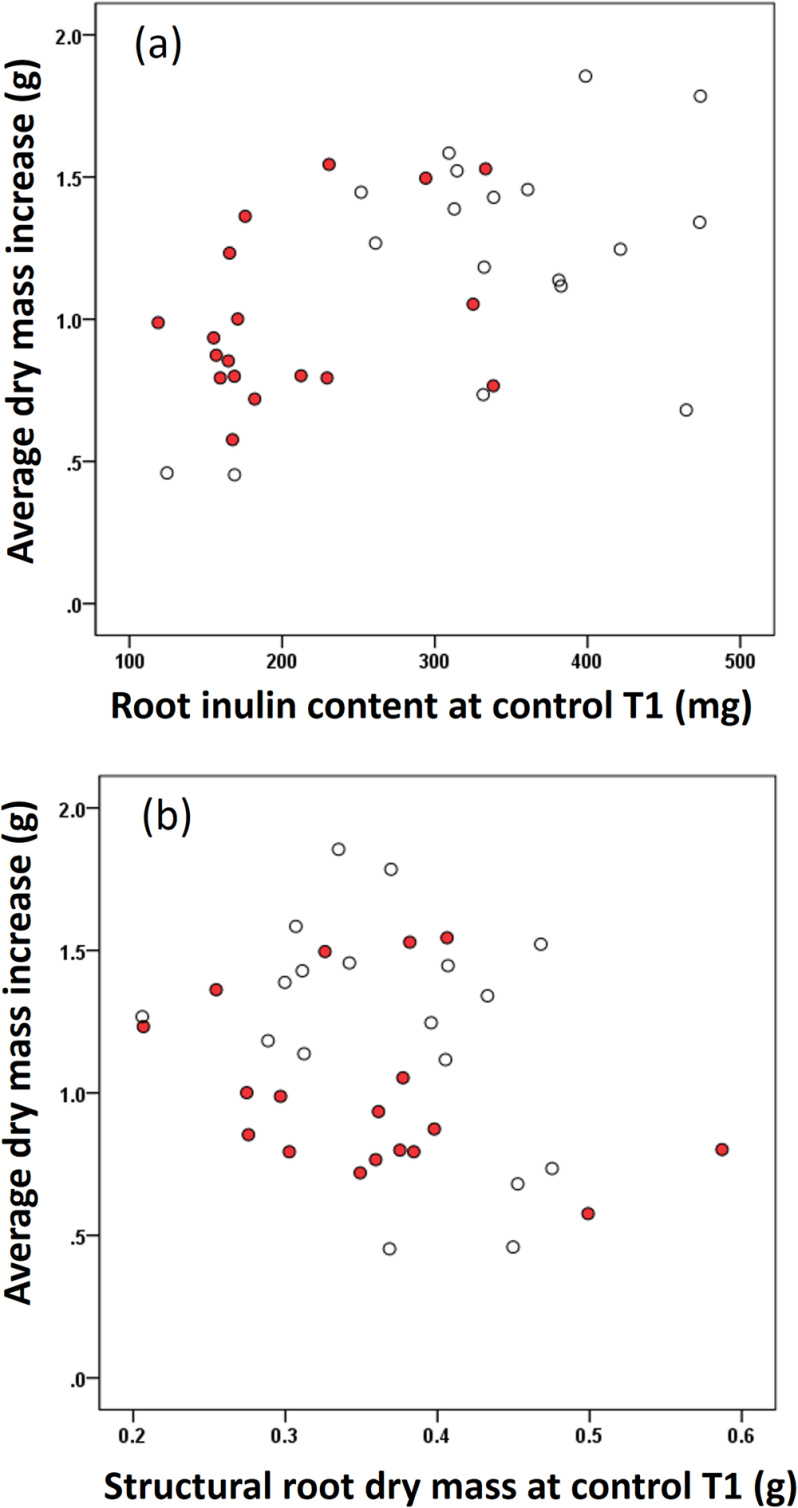
Scatter-plots of the average dry mass increased per population from the defoliation treatment after regrowth against the average root inulin content (**a**) and the average structural root dry mass (**b**) of the population at the moment of defoliation (control T1). Open dots indicate native populations and filled dots indicate invasive populations. Average plant dry mass increase = total dry mass of plants in the defoliation T2- total dry mass of plants in the defoliation T1 (Multiple regression, n = 36, R^2^ = 0.404 full model, total inulin, t = 4.194, p < 0.001; structural root dry mass t = −2.810, p = 0.008). Structural root dry mass is the difference between total root dry mass and root inulin content.

A multiple regression analysis showed that average dry mass (=average total dry mass per population of defoliation T2 minus average total dry root mass of control population T1) increased with the root inulin content of control T1 plants after one month of regrowth (Fig. 4a) and decreased with the structural root mass of the control T1 (Fig. 4b).

The average root inulin content of each population of each harvest was not correlated with the mean temperature of coldest quarter of its corresponding sample site (Fig. S3).

### Parallel evolution

**Figure 5:**
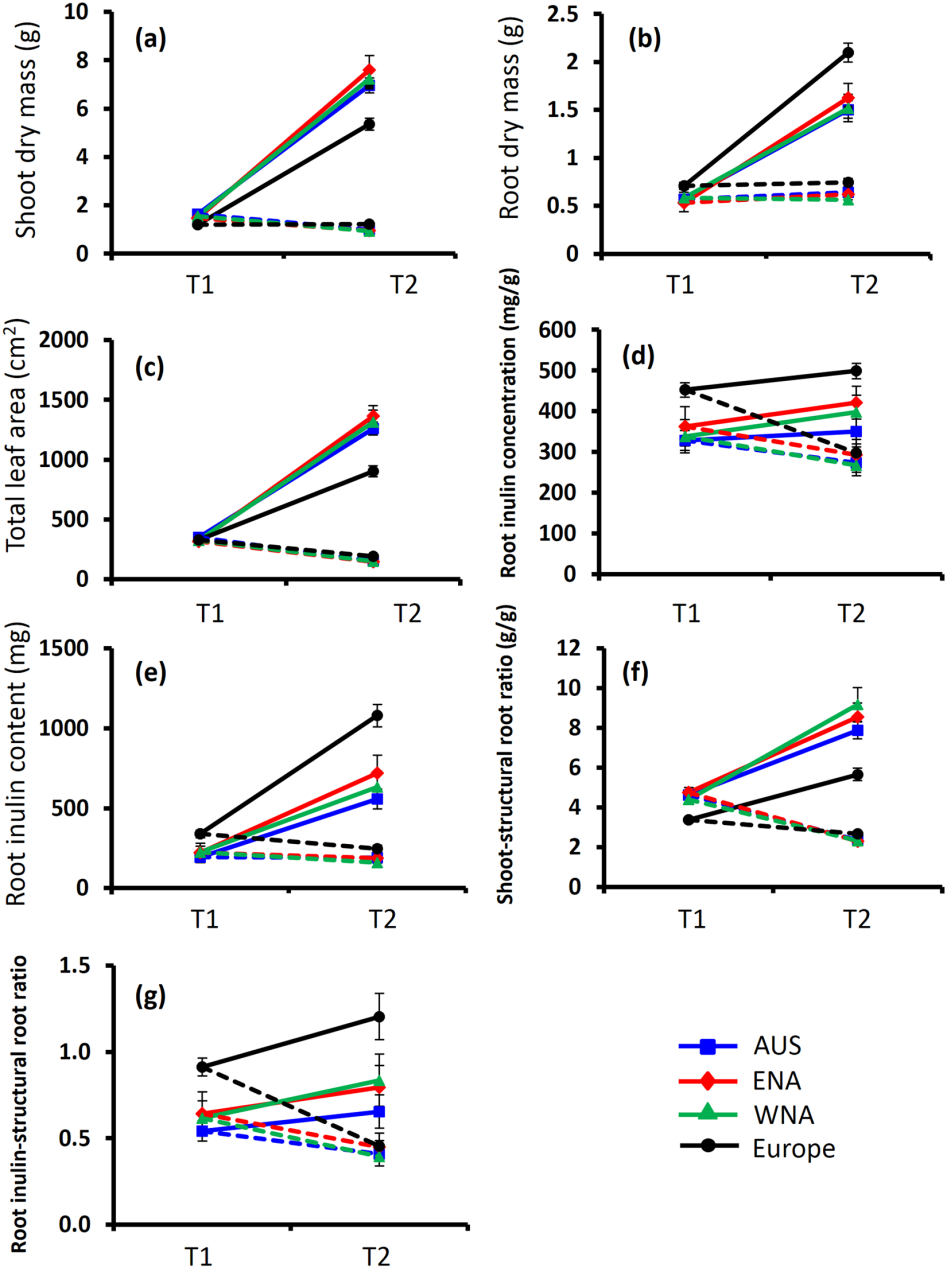
Shoot dry mass (**a**), root dry mass (**b**), total leaf area (**c**), root inulin concentration (**d**), root inulin content (**e**) shoot-structural root ratio (**f**) and root inulin-structural root ratio (**g**) of the invasive populations from three geographic regions (Australia, Western North America and Eastern North America) and native populations from Europe at control T1, control T2 and defoliation T2 respectively. Values are means ± SE. Solid lines correspond to the control treatment and dashed lines are from defoliation treatment.

All studied traits (growth, regrowth ability, root shoot ratios, leaf mass area, etc.) that significantly differed between invasive and native genotypes, changed in the same magnitude and direction for the invasive *J*. *vulgaris* from the three geographically distinct regions (Table 1 and Fig. 5).

## Discussion

Invasive *J*. *vulgaris* genotypes from the three regions grew a larger total leaf area, a heavier shoot mass, a larger total mass and had a higher shoot-structural root ratio than native genotypes in the control treatment at the final harvest at 12 weeks (Control T2, Table 1 and Fig. 2). This was already evident at 8 weeks except for total plant mass (Control T1, Table 1 and Fig. 2). This is in line with the study by Joshi and Vrieling^8^, who, in a common garden experiment, found that after eight months of growth, invasive *J*. *vulgaris* had a higher vegetative growth and a 37% higher reproductive output than native plants. Indeed in several plant species, invasive genotypes have a superior growth ability compared to their native congeners as predicted by the EICA hypothesis^48,49^. However, defoliation of the shoot had reversed the outcome. Therefore we hypothesize that invasive plants trade their regrowth ability for better growth ability. In our study, we found that native *J*. *vulgaris* performed better than invasive genotypes after 4 weeks of regrowth following complete removal of the shoot. Similar results were found by Joshi and Vrieling^8^ who observed higher regrowth of native *J*. *vulgaris* genotypes after complete defoliation when compared with invasive genotypes. Furthermore, Lin, *et al*.^50^ found that native *J*. *vulgaris* genotypes regrew better than invasive genotypes after herbivory by the generalist *Mamestra brassicae* and by the specialist *T*. *jacobaeae* under an intra-specific competition setup. This result indicates that native *J*. *vulgaris* are better adapted to complete defoliation than invasive ones. Apparently this comes at the cost of a reduced growth rate because in the invasive areas where *J*. *vulgaris* does not have to cope with severe defoliation by specialist herbivores they have lost part of their regrowth ability but attain higher growth. In contrast, other studies found that invasive Chinese tallow tree (*Sapium sebiferum*) genotypes have higher regrowth ability than native genotypes after both simulated and natural herbivory^51,52^. It was argued that fast regrowth may be an important defense against local generalist herbivory after invasion. Higher regrowth ability might also contribute to the invasion success, especially after disturbances such as mowing, fire and grazing^53^. In the invasive range, *J*. *vulgaris* mostly occurs in extensively grazed pastures, where it is avoided by cattle that are repelled by the toxic pyrrolizidine alkaloids. In the invasive range, *J*. *vulgaris* genotypes produce more toxic pyrrolizidine alkaloids, which could effectively defend them against grazers and other local generalist herbivores^8^. Ragwort does occur in temperate climates in the invasive ranges which are less likely to be disturbed by fire. It suggests that the role of disturbances on regrowth capacity by grazing and fire in the invasive range are limited. Storage in roots is also needed to survive cold winters^35^. Here we found that, on average, the mean temperature of the coldest quarter in the sampled invasive populations was equal to that in the native range and that storage was not correlated with the mean temperature of the coldest quarter. It suggests that the decrease in carbohydrate storage in *J*. *vulgaris* is not related to the colder winters in the invasive ranges.

The regrowth ability we estimated, reflects the fraction biomass in root and how well plants can compensate for its aboveground loss. In general, native *J*. *vulgaris* genotypes have relatively more biomass in the roots, so a smaller fraction of aboveground biomass is removed at the moment of defoliation which gives these plants an advantage at the beginning of regrowth. Native genotypes also stored more inulin in their roots than invasive genotypes. Our data showed that the increase in biomass after regrowth is positively correlated with the root inulin content at the moment of defoliation, and negatively correlated with the size of the structural root at the moment of defoliation (Fig. 4). Several studies, mainly on grasses, have reported that the root carbohydrate storage is positively associated with plant regrowth after defoliation^23,54,55^. Furthermore, Danckwerts and Gordon^56^ used isotopes to confirm that stored root carbohydrates in the grass *Lolium perenne* have been remobilized to the newly regenerated leaves in response to defoliation. Our results also show that the regrowth performance of *J*. *vulgaris* depends on root inulin storage. It suggest that invasive *J*. *vulgaris* in the absence of its main specialist herbivore *T*. *jacobaeae* have been selected to produce smaller roots and store less inulin resulting in poorer regrowth ability. Our study is the first that shows a positive correlation between root carbohydrate storage and regrowth ability for invasive plants, in comparison to native plants which had greater root carbohydrate storage and greater regrowth ability.

On the other hand, the allocation of large amounts of energy to carbohydrate storage in the roots seem to come at the cost of decreased growth. It suggests that the carbohydrates stored in the roots cannot be converted into photosynthetic tissue, and therefore incur a loss in growth rate. Invasive *J*. *vulgaris* genotypes, which allocate less to carbohydrate storage, showed significantly more biomass accumulation than native genotypes (Table 1). It suggests that regrowth is a costly strategy.

Furthermore, we found that the plant biomass after regrowth, decreased with structural root dry mass (Fig. 4). It indicates that the maintenance of the structural root cells is costly and that energy is needed to keep these cells alive and functioning^28,29^. Therefore a bigger structural root, without a photosynthesizing shoot, consumes a bigger amount of the stored carbohydrates. We found that the size of structural roots is negatively correlated with plant regrowth ability after defoliation. In general, previous studies have considered the ratio between total root and total shoot as an indicator of storage or regrowth^9,10^. Here we find that a large root does not necessarily translate into better regrowth ability, but that size of the root may even negatively affect plant regrowth after damage. We show that it is crucial to study the root storage and structural components separately rather than associating the entire root with plant regrowth ability.

A model proposed by de Jong and van der Meijden^57^ suggested that under repeated disturbance such as the outbreak of a specialist herbivore, plant genotypes that allocate more resources to storage are better adapted to herbivory and recover quickly through regrowth since a smaller fraction of their total biomass is removed by herbivory. Due to herbivory, plants have been selected to evolve different allocation strategies and store more resources in the roots. We found that the root carbohydrate storage in native *J*. *vulgaris* counts for 50% of the root biomass while other herb species often contain less (ca. 10%~20%) carbohydrate storage^58,59^. The presence of the specialist *T*. *jacobaeae* larvae in the native range may explain why native *J*. *vulgaris* developed increased carbohydrate storage. The larvae of this univoltine specialist herbivore have broken through all plant defenses and *J*. *vulgaris* uses regrowth as a strategy to overcome defoliation^9,41^. It suggests that the lack of selection pressure by the specialist herbivore *T*. *jacobaeae* in the invasive range plays an important role in the evolution of a decreased regrowth ability of invasive *J*. *vulgaris*. The foliar-feeding larvae of this specialist herbivore regularly defoliate all the aboveground parts of *J*. *vulgaris* in the native range during June^41,60,61^. We show that the higher shoot-structural root ratio in the non-defoliated invasive *J*. *vulgaris* genotypes in our experiment represents a redistribution of resources from root storage to aboveground parts, thus contributing to a better growth performance.

Our results further showed that, of all the studied traits that significantly changed in invasive *J*. *vulgaris* genotypes, all changed in the same magnitude and direction in the three geographically distinct regions (Fig. 5). Genetic analyses showed that multiple introductions most likely occurred in the invasive ranges^39^ and this strongly implies that the changes in these traits can be best explained by a parallel evolution in the invasive *J*. *vulgaris* genotypes. Similar results were found for the competitive ability of invasive *J*. *vulgaris*. Since the major abiotic factor, local climatological conditions, are significantly different among the three invasive regions, such a parallel evolution is more likely due to the release from specialist herbivores than to adaptation to local abiotic factors after invasion.

In conclusion, in this study we found that invasive *J*. *vulgaris* genotypes have been selected to evolve increased growth ability and decreased regrowth ability compared to native genotypes, due to the lower investment in root carbohydrate storage. These results support the EICA and the SDH, which contend that due to the absence of adapted specialist herbivores, a net gain is obtained for increasing growth by the invasive plants as they invest less in anti-herbivore defense. It shows that plant regrowth ability after defoliation is strongly correlated with carbohydrate storage in the root while the size of the structural root is negatively correlated with regrowth ability. Additionally, all the studied traits measured in the invasive *J*. *vulgaris* genotypes from the three geographically distinct regions changed in the same direction suggesting a parallel evolutionary response to the absence of specialist herbivores.

## Materials and Methods

### Plant material and growth conditions

To sample genetic variation as broadly as possible, seeds were collected from 18 native populations in Europe and 18 invasive populations in Australia, Western and Eastern North America (for detailed population information see Supporting Information Table S1 and Fig. S1). Native seeds from the potential source populations along the western coast of Europe were selected to compare them with invasive populations^39^. For each population, seeds from three different plants (hereafter referred to as “mother plants”) were germinated in petri dishes with moistened filter paper. After 5 weeks, 4 well-grown seedlings from each mother plant were selected and randomly assigned to the control and defoliation treatments and plants were harvested at control T0 (seedling harvested at T0 before potting), control T1 (plants harvested at T1), control T2 (plants harvested at T2) and defoliation T2 (Plants defoliated at T1 and harvested at T2 (Fig. S2). Therefore each of the 4 treatments contained 108 plants (1 seedling * 3 mother plants * 36 populations). Seedlings from control T0 were harvested and oven-dried for 3 days at 50 °C and then weighed. Plants from control T1, T2 and defoliation T2 were potted into 1 L pots with 20% potting soil (Slingerland potting soil, Zoeterwoude, The Netherlands), 80% dune sand (collected from Meijendel, The Netherlands, 52°13′N, 4°34′E) and 2.5 g Osmocote slow-release fertilizer (Scott®, Scotts Miracle-Gro, Marysville, Ohio, USA; N:P:K:MgO 15:9:11:2.5). Plants were grown in a climate room at 20 °C, 70% humidity, 16 hours daylight with a light intensity of 113 umol PAR m^−2^ s^−1^. Eight weeks after potting, plants from control T1 were harvested and plants from the defoliation T1 were defoliated by removing the shoot (1 cm above the root crown) with a pair of scissors. The plants used were rosettes so the defoliation treatment removes only leaves as the flowering stem is not yet present and the growing point was left intact. The removed shoot materials were dried in an oven at 50 °C for three days and weighed to determine their dry mass.

Subsequently, plants from control T2 and defoliation T2 were allowed to grow for a further 4 weeks (from T1 to T2) till their final harvest after which their dry mass was determined. The samples were dried as described previously and the dry mass of shoots and roots were determined. Except for control T0, the total leaf area of each plant from the other three treatments was measured at harvest using a portable leaf area meter (LI-3100, LICOR, Inc., Lincoln, NE, USA). Two invasive plants from control T1, 2 invasive and 4 native plants from defoliation T2 and two native plants from control T2 were dead during the experiment and were not harvested.

### Root inulin concentration (Carbohydrate storage)

Inulin, a fructose polymer, is the carbohydrate storage compound in roots of the *Asteraceae*^62^. The concentration of root inulin was measured as the difference between the free sugar content before hydrolysis and the total sugar content after hydrolysis with inulinase. For each plant, 0.1 g of finely powdered root material was incubated with 4 mL distilled water at 80 °C for 1 hour to determine the free sugars. After centrifugation, 1 ml of the supernatant was mixed with 2 mL of 0.03 M 3, 5-dinitrosalicylic acid (DNS). The absorbance of this solution was recorded with a spectrophotometer at 540 nm and the concentration of free sugar was calculated using a calibration curve made with D(−) fructose according to Miller^63^. Another 1 mL of the same sample was hydrolysed with 200 µl of inulinase (Novozym®960, Sigma-Aldrich) for 1 hour at 60 °C and the concentration of total sugar was measured as above. The total inulin content (mg) in the root was calculated [=root inulin concentration (mg•g^−1^) × root dry mass (g)]. Analysis of the inulin content in the shoot showed that it was less than 10% of that of the root and that it did not differ between native and invasive genotypes (Data not shown).

Since roots are used for carbohydrate storage, to maintain structure, and for acquiring nutrients and water, we divided the total root dry mass into the carbohydrate storage root dry mass (=root inulin content) and the structural root dry mass (=total root dry mass − root inulin content). Additionally, we calculated the root inulin-structural root ratio (=root inulin dry mass in the root/structural root dry mass) and shoot to structural root ratio (=shoot dry mass/structural root dry mass). The plant regrowth ability was estimated as the ratio between the total dry mass of defoliation T2 and the total dry mass of control T2 according to van der Meijden, *et al*.^43^.

### Climatic conditions

To examine the difference in the local climate among the four geographic regions (Europe, Australia, Western and Eastern North America), all the available 19 bioclimatic variables (ca 1950–2000) were downloaded from the World Clim dataset (http://www.worldclim.org/current) in 5 arc-minutes resolution for each sampled population. A principal components analysis (PCA) was performed with the SIMCA-P software (v.11.0, Umetrics, Umeå, Sweden) to classify all sampled populations based on the 19 bioclimatic variables. The scaling method for the PCA was unit-variance.

### Statistical analysis

As the main interest of this study was to find the differences among the native and three invasive regions (Australia, West and Eastern North America), a nested ANOVA with region as a fixed factor and population nested within region as a random factor combined with Tukey HSD post-hoc test were performed for all the measured traits in all the three treatments. Normality of the residuals was tested with a Kolmogorov-Smirnov test. To obtain normality, shoot-structural root ratio and root storage-structural root ratio were log transformed. Furthermore, homogeneity of variance in the ANOVA was tested with Levene’s test and in all cases this test was non-significant.

We tested the shoot dry mass at T1 (6 motherplants for each population; 3 from defoliation T1 and 3 from control T1), and found significant differences among populations (ANOVA, df = 1, 35; p = 0.02). We therefore used the population average to correlate the initial root inulin content and the structural root dry mass at the moment of defoliation with the net gain in dry mass one month after regrowth. A multiple regression analysis was conducted using the average dry mass increase per population (=average total dry mass per population in the defoliation T2- average root dry mass per population in the defoliation T1) as the dependent variable and the average total inulin content and the average structural root dry mass from each population at defoliation T2 as independent variables.

Since inulin might play an important role for plants to survive the winter, we correlated the average root inulin content per population for each harvest with the mean temperature of the coldest quarter of that population. All analyses were carried out using SPSS 18.0 (SPSS: An IBM Company, Chicago, USA).

## Electronic supplementary material

SUPPORTING INFORMATION
