# Supplementary material for: Evolutionary changes in growth, regrowth and carbohydrate storage in an invasive plant
Source: Sci Rep. 2018 Oct 8;8:14917. doi: 10.1038/s41598-018-33218-z (PMC6175876; doi:10.1038/s41598-018-33218-z)
Supplement: Supplementary file 1 — SUPPORTING INFORMATION [file 41598_2018_33218_MOESM1_ESM.docx]

Supplementary Information

Manuscript title: Evolutionary changes in growth, regrowth and carbohydrate storage in an invasive plant

Authors: Tiantian Lin, Peter G.L. Klinkhamer, Klaas Vrieling


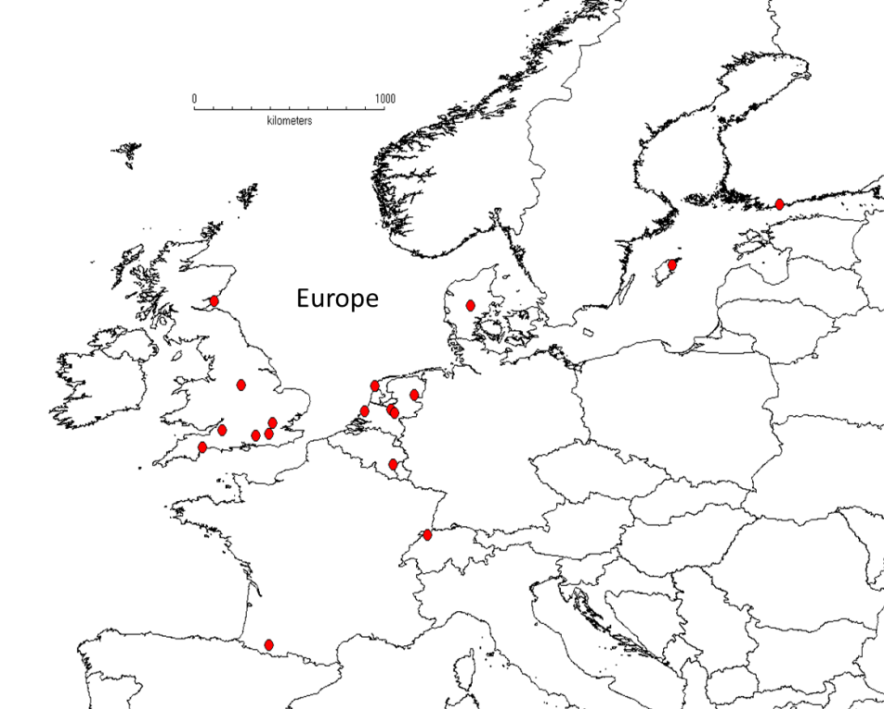


(a)


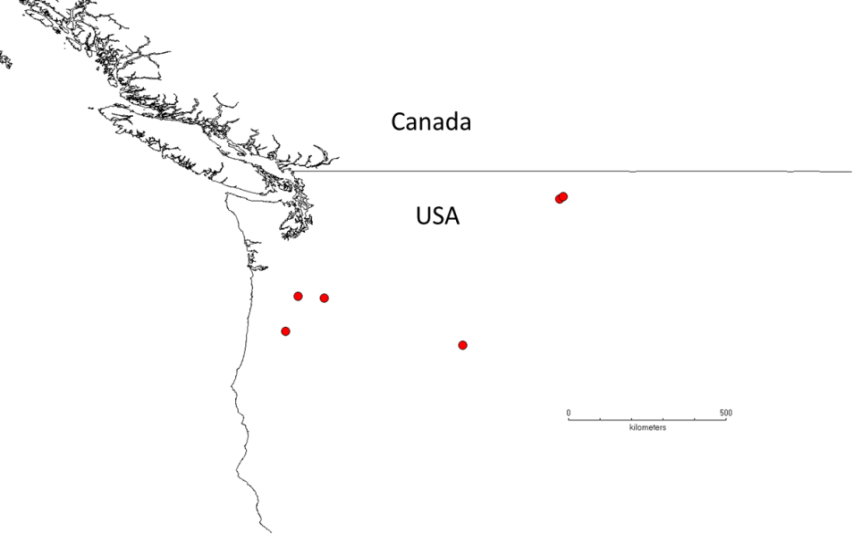


(b)


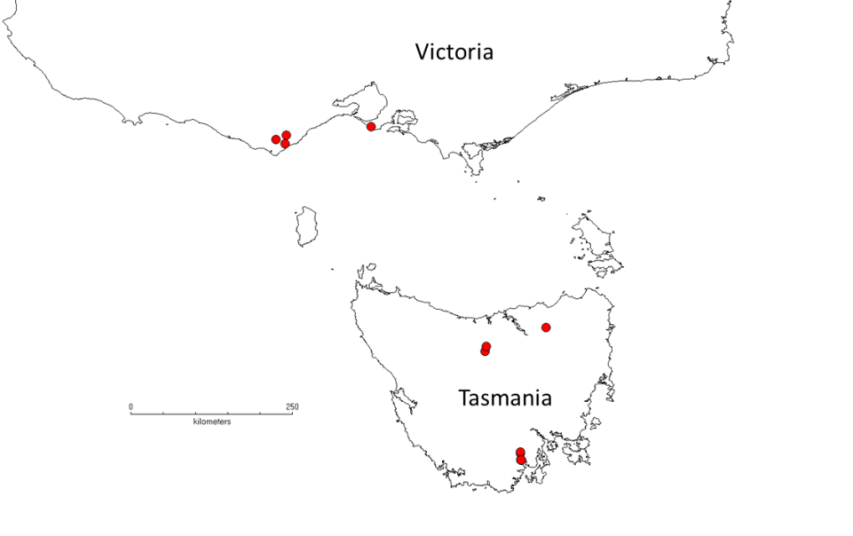


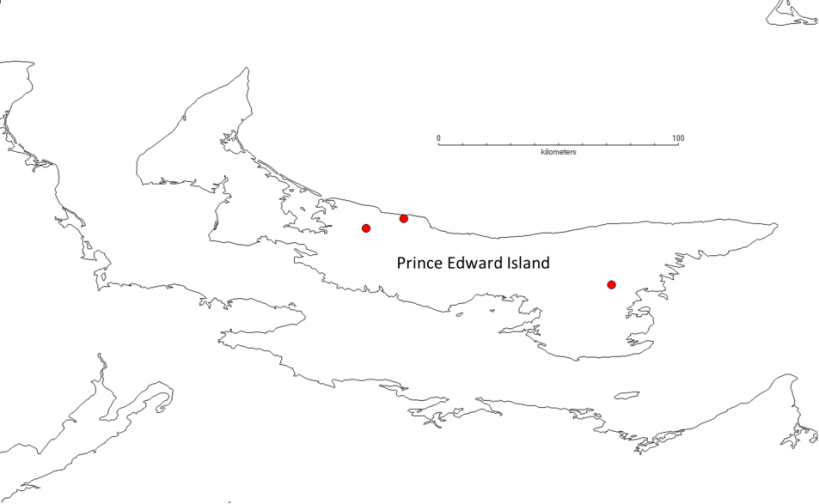


(d)

(c)

Supplementary Figure 1 | Distribution map of native and invasive *J. vulgaris* populations used in this study from Europe (a, n=18 populations), Western North America (b, n=6 populations), Eastern North America (c, n=3 populations) and Australia (b, n=9 populations)


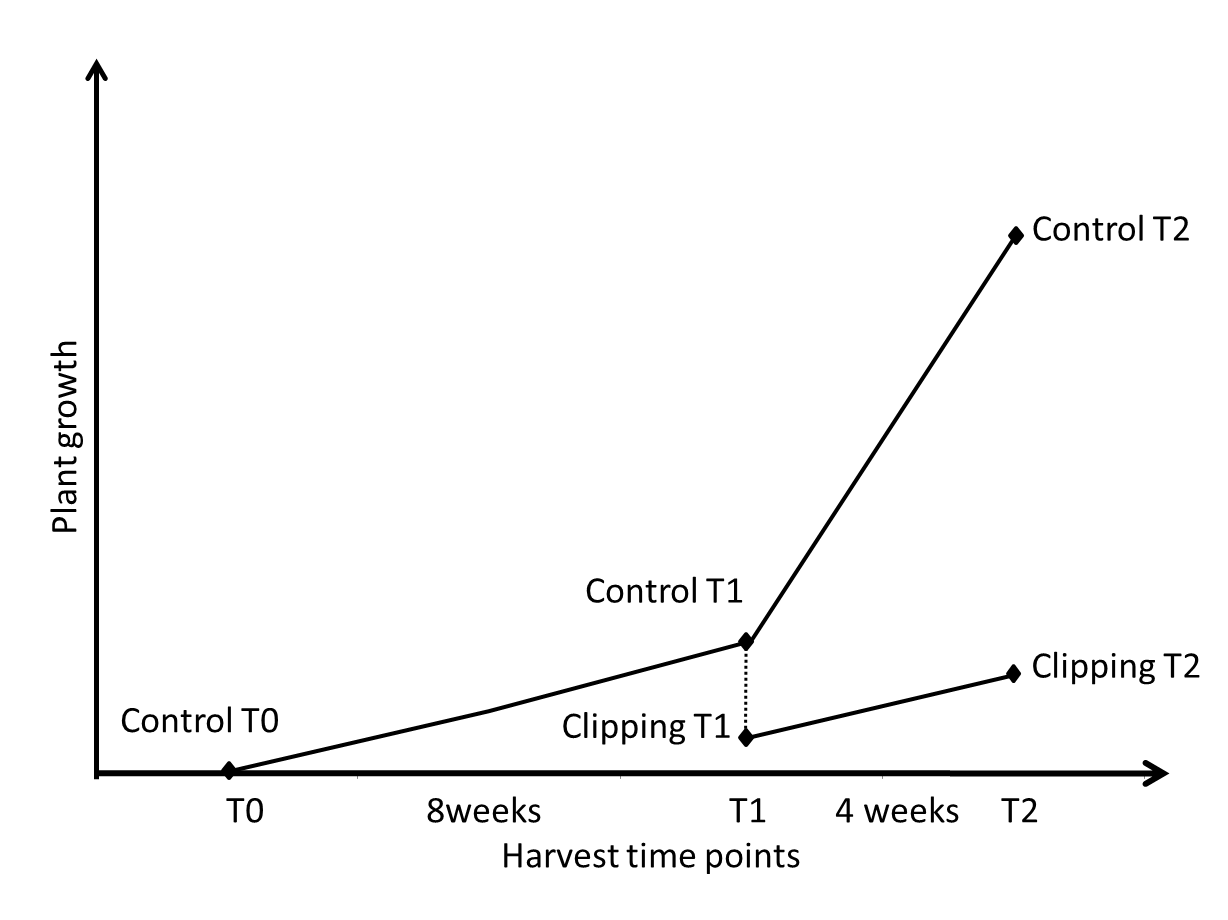


Supplementary Figure 2 | Experimental design for the control treatment and the clipping treatment and the harvest time points in this study. For the control treatment, plants were harvested at T0, T1 and T2. For the clipping treatment, plant shoots were clipped at T1 and plants were harvested at T2.

Supplementary Table 1 | Origin of seeds of 18 native and 18 invasive populations of *Jacobaea vulgaris* used in this study

| Origin | country | Location | Longitude | Latitude |
| --- | --- | --- | --- | --- |
| *Invasive* | Australia | Franklin, Tasmania | 146°19' E | 41°33' S |
|  |  | Dairy Plains, Tasmania | 146°31' E | 41°38' S |
|  |  | Wild Dog Road, Victoria | 143°40' E | 38°41' S |
|  |  | Beech Forest, Victoria | 143°33' E | 38°38' S |
|  |  | Barramunga, Victoria | 143°41' E | 38°34' S |
|  |  | Cape Schanck, Victoria | 144°54' E | 38°27' S |
|  |  | Dairy Plains, Tasmania | 146°32' E | 41°34' S |
|  |  | Targa, Tasmania | 147°23' E | 41°18' S |
|  |  | Franklin, Tasmania | 147°01' E | 43°04' S |
|  | Eastern North America | Cardigan, Prince Edward Island | 63°37' W | 46°13' N |
|  |  | Cavendish Beach, Prince Edward Island | 63°24' W | 46°29' N |
|  |  | Clinton, Prince Edward Island | 63°32' W | 46°26' N |
|  | Western North America | Indian Creek, Oregon | 117°49' W | 44°01' N |
|  |  | West Crestmont, Oregon | 121°51' W | 45°22' N |
|  |  | Island lake, Oregon | 122°37' W | 45°25' N |
|  |  | Island Lake Road, Montana | 114°59' W | 48°20' N |
|  |  | Kootenai National Forest, Montana | 114°53' W | 48°17' N |
|  |  | Cochran Creek, Oregon | 122°58' W | 44°25' N |
| *Native* | Belgium | Bertogne | 05°40' E | 50°05' N |
|  | Denmark | Engesvang | 09°21' E | 56°10' N |
|  | Finland | Inkoo, Kirkkonummi | 24°00' E | 60°02' N |
|  | France | Lourdes | 00°13' W | 43°09' N |
|  | The Netherlands | Meijendel | 04°20' E | 52°07' N |
|  |  | Wageningen | 05°34' E | 52°10' N |
|  |  | Mossel | 05°45' E | 52°03' N |
|  |  | Gees | 06°41' E | 52°44' N |
|  |  | Texel | 04°48' E | 53°05' N |
|  | Sweden | Sit Olofsholm, Gotland | 18°54' E | 57°43' N |
|  | Switzerland | Mettembert | 07°19' E | 47°23' N |
|  | UK | Alice holt forest | 00°50' W | 51°10' N |
|  |  | Saint Andrews | 02°47' W | 56°20' N |
|  |  | Derbyshire | 01°30' W | 53°07' N |
|  |  | Sevenoaks weald | 00°12' W | 51°14' N |
|  |  | Marshgreen | 03°21' W | 50°44' N |
|  |  | Enfield | 00°03' W | 51°40' N |
|  |  | Corston | 02°26' W | 51°23' N |
